# Exploiting the Potential of Biosilica from Rice Husk as Porous Support for Catalytically Active Iron Oxide Nanoparticles

**DOI:** 10.3390/nano11051259

**Published:** 2021-05-11

**Authors:** Ana Franco, Rafael Luque, Carolina Carrillo-Carrión

**Affiliations:** Departamento de Química Orgánica, Campus de Rabanales, Universidad de Córdoba, Ed. Marie Curie, Ctra. Nnal. IV-A, Km 396, E14014 Córdoba, Spain; b12frloa@uco.es

**Keywords:** rice husk, silica, mechanochemistry, iron oxide nanoparticles, hematite phase, heterogeneous catalysis

## Abstract

Biomass-derived materials are put forward as eco-friendly alternatives to design heterogeneous catalysts. To contribute in this field, we explored the potential of mesoporous biogenic silica (RH-Silica) obtained from lignocellulosic waste, in particular from rice husk, as an inorganic support to prepare heterogenized iron oxide-based catalysts. Mechanochemistry, considered as a green and sustainable technique, was employed to synthetize iron oxide nanoparticles in pure hematite phase onto the biosilica (α-Fe_2_O_3_/RH-Silica), making this material a good candidate to perform catalyzed organic reactions. The obtained material was characterized by different techniques, and its catalytic activity was tested in the selective oxidation of styrene under microwave irradiation. α-Fe_2_O_3_/RH-Silica displayed a good catalytic performance, achieving a conversion of 45% under optimized conditions, and more importantly, with a total selectivity to benzaldehyde. Furthermore, a good reusability was achieved without decreasing its activity after multiple catalytic cycles. This work represents a good example of using sustainable approaches and green materials as alternatives to conventional methods in the production of high-added value products.

## 1. Introduction

Nanomaterials have witnessed an explosion in the last decades due their extraordinary properties, which make them rather versatile and suitable to be applied in many scientific fields [1,2,3]. In the global context of climate change emergency, great efforts are being made to develop more sustainable and efficient nanocatalysts for the production of materials, fine chemicals and energy [4,5]. Among them, nanostructured inorganic materials, such as metal nanoparticles (MNPs), are an alternative to conventional metallic homogeneous catalysts, due to their superior properties (e.g., quantum effects due to the reduction in size, and surface effects due to the large surface areas) in comparison to their bulk counterparts [6]. However, they are not thermodynamically stable and difficult to recycle due to their tiny size (required to be highly active), which are critical issues for their application in green catalysis [7]. To overcome their deactivation due to aggregation and to facilitate their posterior recovery from the reaction media, nanoparticles (NPs) must be immobilized on supports [6,8]. Traditionally, inorganic materials, such as alumina [9,10], silica [11], zeolites [12,13], or titania [14] have been used as supports in the design of heterogeneous catalysts; however, due to environmental concerns, alternative supports that are more environmentally friendly and sustainable are highly desirable. In this direction, biomass can play a crucial role since today it is well known the possibility of obtaining a handful of nanomaterials with potential to act as supports using biomass and residues as starting materials [15,16,17].

Lignocellulosic biomass, which comprises the cell walls of plants, is a renewable feedstock composed mainly by three biopolymers (i.e., cellulose, hemicellulose, and lignin), together with small amounts of inorganic material (ashes) [18]. Due to its composition, biomass can be transformed by multiple approaches to produce energy, or useful materials [19,20]. In this context, food industry produces tons of waste [21] without any economic value, which are further eliminated through contaminant and expensive processes. Rice husk (RH), an agricultural waste produced during the industrial rice processing, has received a great deal of attention thanks to its high silica content (20 wt%), being thus considered as a valuable and sustainable source to obtain silica-based materials [15,22]. Based on these premises, our group have developed an integrated mechanochemical/microwave-assisted methodology to obtain highly pure mesoporous biogenic silica (RH-Silica) from rice husk [23], which is exploited in this work as an effective porous inorganic support. 

On the other hand, many organic reactions catalyzed by nanomaterials pursue the goal of achieving selectivity towards the desired product (being sometimes challenging to get a 100% selectivity), while maintaining good yields. Oxidation of alkenes is one of the most important transformation in the production of valuable chemicals for food, pharmaceutical, and fine industries; unfortunately, over-oxidations are a typical issue that leads to a limited selectivity [24,25]. Among the different possible compounds (e.g., aldehydes, epoxides, or acids) [26,27], benzaldehyde has a particularly high relevance due to its wide use in industry [28,29]. Nowadays, the industrial production of benzaldehyde is mainly achieved by two processes: (i) the hydrolysis of benzal chloride, and (ii) the oxidation of toluene to benzoic acid [30,31]. These routes, however, have important drawbacks, such as the need of high temperature, long reaction times, and/or the production of undesirable wastes [32], which make these procedures far from being sustainable. In the search for alternative synthetic routes, the oxidation of styrene has emerged as an eco-friendly way to produce benzaldehyde; therefore, developing efficient and sustainable heterogeneous catalysts to promote this reaction with good yields and selectivity is of great importance. In this context, transition metal oxides are an alternative to the precious-metal-based catalysts [33] and among them, iron oxide nanoparticles are promising low-cost and eco-friendly catalysts to perform multiple organic reactions, such as oxidations, alkylations, redox reactions, and C–C couplings reactions [34,35,36,37,38]. 

**Scheme 1:**
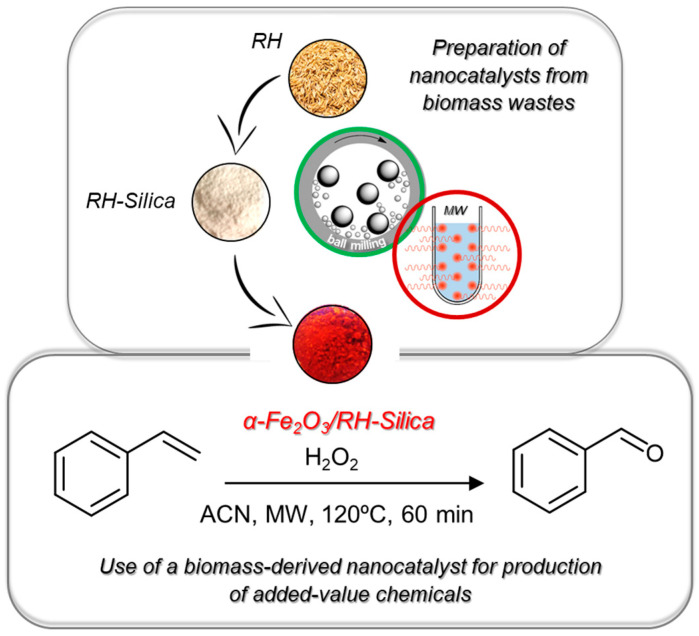
Preparation and catalytic application of the α-Fe_2_O_3_ /RH-Silica material starting from rice residues by combining mechanochemistry and microwave techniques.

Herein, exploiting the potential of mesoporous biogenic silica as inorganic porous support, we performed the in-situ synthesis and immobilization of catalytic iron oxide NPs with a precise control in their crystalline phase (hematite, α-Fe_2_O_3_) by means of a mechanochemistry approach. The applicability of the as-synthetized catalyst (α-Fe_2_O_3_ /RH-Silica) was then tested in the selective microwave-assisted oxidation of styrene to benzaldehyde using hydrogen peroxide as green oxidizing agent, cf. Scheme 1.

## 2. Results and Discussion

**Figure 1:**
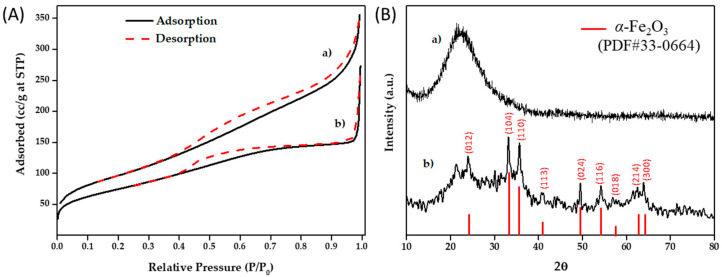
(**A**) N_2_ physisorption isotherms obtained from a) RH-Silica and b) α-Fe_2_O_3_/RH-Silica materials. (**B**) XRD patterns of (a) RH-Silica and (b) α-Fe_2_O_3_ /RH-Silica; XRD peak lines from standard powder diffraction files of hematite α-Fe_2_O_3_ (PDF#33-0664) are shown as red lines with the corresponding crystal planes.

Rice husk biosilica (RH-Silica) was obtained by following a microwave/mechanochemistry approach, developed previously by our group and patented [23,39]. RH was milled, microwave-assisted digested, and the solid obtained was then washed and calcinated to obtain a highly pure mesoporous biosilica. RH-Silica was used as an inorganic support to design an acid iron oxide nanoparticles-based catalyst (Fe_2_O_3_/RH-Silica). Iron oxide NPs were efficiently deposited onto the RH-Silica surface by mechanochemistry using an iron salt as a precursor together with propionic acid. The propionic acid serves as complexing agent to form the Fe-propionate complex, which is further decomposed thermically to obtain the NPs, as well as “anchoring” agent to favor the incorporation of the iron in the support. Without the addition of this carboxylic acid the iron loading was demonstrated to be much lower. Additionally, it may provide a weak reducing environment to protect divalent iron salts from oxidation, and thus facilitate the formation of a pure hematite crystalline phase. N_2_ physisorption isotherms obtained for both materials (Figure 1A) correspond to type IV, which is characteristic of mesoporous materials. RH-Silica presented a surface area of 352 m^2^·g^−1^ and a pore size and pore volume of 8.0 nm and 0.56 cm^3^·g^−1^, respectively (Table 1). After the mechanochemical process, Fe_2_O_3_/RH-Silica exhibited a lower surface area 272 m^2^·g^−1^ due to the iron oxide NPs incorporation. Comparably, the pore size and the pore volume also decreased (7.1 nm; 0.39 cm^3^·g^−1)^ as a consequence of a minor structure collapsing and a partial migration of the NPs into the pores.

**Figure 2:**
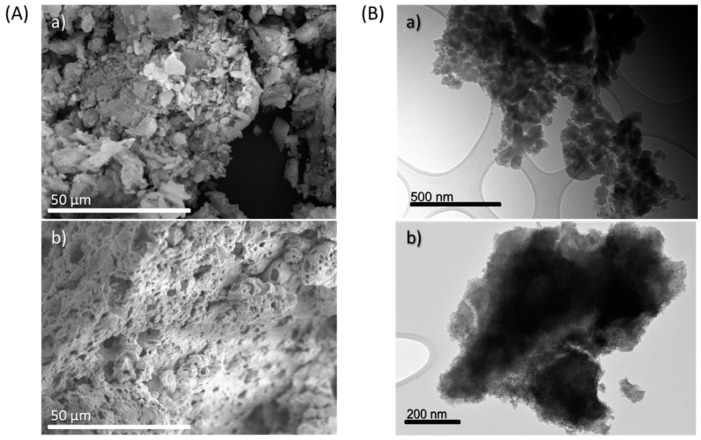
(**A**) SEM and (**B**) TEM images of (a) RH-Silica and (b) α-Fe_2_O_3_/RH-Silica.

The structure of the nanomaterials was also studied by X-Ray Diffraction (XRD, Figure 1B). RH-Silica diffractogram showed a broad peak at 2θ = 22–25° which corresponds to the amorphous silica phase. As expected, this peak decreased its intensity after the incorporation of the NPs, whereas the characteristic peaks of iron oxide in hematite phase (α-Fe_2_O_3_) appeared [40]. Note that all peaks of the XRD patterns are matched with the phase of α-Fe_2_O_3_ (PDF#33-0664), without any peak associated to other iron oxide forms such as magnetite (Fe_3_O_4_) and maghemite (γ-Fe_2_O_3_). It is noteworthy that both (i) high localized temperatures achieved during the mechanochemical/milling process, and (ii) the following thermal treatment at 300 °C, are likely key factors for yielding the NPs in pure hematite phase (α-Fe_2_O_3_). Magnetite (Fe_3_O_4_) is a metastable oxide and Fe^2+^ is easily oxidized to Fe^3+^ even under ambient conditions, resulting in maghemite (γ-Fe_2_O_3_), which is further transformed to the hematite phase at temperatures ≥ 300 °C [41,42]. However, the transition temperatures between phases should be taken with caution since it is known that the particle size affects strongly to the changes in the crystalline structure; the smaller the NP is, the lower the temperature required for a phase transition will be [43]. One major advantage of having Fe_2_O_3_ NPs in hematite phase for catalytic applications is its superior thermal and chemical stability. This allows performing reactions at harsh conditions but maintaining the structural integrity of the catalyst, which is an essential feature for their further reusability. Inductively coupled plasma mass spectrometry (ICP-MS) analyses were performed to confirm the successful incorporation of the Fe species as well as to quantify the Fe doping level, which resulted to be ca. 22 wt% (21.3 wt% and 23.5 wt% for two different batches), showing thus a high reproductivity of the synthetic procedure. It is worth noting that this value indicates a quite good loading efficiency by taking into account that the theoretical maximum loading content (total Fe content added in the synthesis) was 37 wt%. Next, Scanning Electron Microscopy (SEM) and Transmission Electron Microscopy (TEM) were employed to study the morphology of the as-prepared nanomaterials. As can be observed in SEM images (Figure 2A) RH-Silica had a typical amorphous and mesoporous structure which drastically changed to a more compact after the incorporation of Fe_2_O_3_ NPs. This may be due to use of propionic acid in the mechanochemical process, which is chemisorbed onto the surface of the Fe_2_O_3_ NPs and serves as an anchoring and stabilizing agent to prevent the leakage of NPs from the support. Furthermore, TEM images (Figure 2B) revealed a no order structure and uniform morphology in the case of RH-Silica, whereas α-Fe_2_O_3_/RH-Silica had a higher density and contrast, which is a clear indication of the successful incorporation of iron oxide NPs to the silica. Unfortunately, the low resolution of the TEM instrument did not make possible the visualization of iron oxide NPs as previously reported in similar materials prepared by the group but characterized by using a high-resolution transmission electron microscopy (HRTEM) [44].

**Figure 3:**
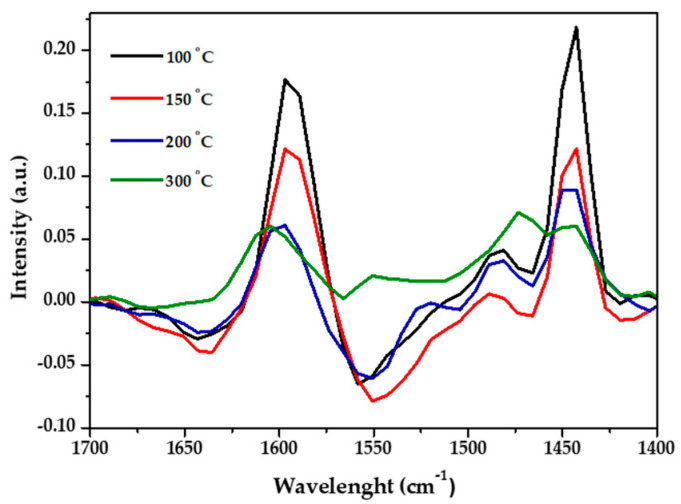
DRIFT spectra of pyridine adsorption at different temperatures on the α-Fe_2_O_3_/RH-Silica material.

To further investigate the surface acidity of the materials, diffuse reflectance infrared Fourier transform spectroscopy (DRIFT) analyses were performed (Figure 3). RH-Silica pyridine adsorption spectrum (not shown) did not exhibit any signal, whereas the spectrum of pyridine adsorbed onto α-Fe_2_O_3_/RH-Silica presented two strong bands at 1442 and 1597 cm^−1^ corresponding to Lewis acid sites. Although the intensity of these bands decreased with temperature as expected, they were still detected at 300 and 200 °C, which clearly points out the potential of α-Fe_2_O_3_/RH-Silica to catalyze Lewis acid-promoted reactions even when high temperatures are required.

The oxidation of styrene has a special relevance in industry, and thus designing more active and selective heterogeneous catalysts is always desirable. For this reason, the oxidation of styrene assisted by microwave irradiation was selected as a model reaction to evaluate the catalytic performance of the as-prepared materials.

After material characterization, RH-Silica and Fe_2_O_3_/RH-Silica were tested for the microwave-assisted oxidation of styrene. The styrene oxidation reaction did not exhibit any conversion in absence of catalyst, same as RH-Silica did not promote any effect in the reaction (Table 2, entries 1 and 2). After the addition of the catalyst, α-Fe_2_O_3_/RH-Silica, it was observed a moderate styrene conversion (45%) and an excellent selectivity to benzaldehyde (>99%) without detecting any other by-product (Table 2, entry 3) under optimized reaction conditions. To optimize the performance of the catalytic reaction we tested different reaction conditions; the parameters studied were the H_2_O_2_ concentration (ranging from 0.1 to 0.5 mL of a 50% *v*/*v* solution), the temperature (in the range of 60–150 °C), and the time (from 10 min to 90 min). The best results (higher conversion and selectivity) were achieved for 0.3 mL H_2_O_2_ (50% *v*/*v*), 120 °C, and 60 min. The increase in the H_2_O_2_ amount led to higher conversions of styrene up to 0.3 mL H_2_O_2_ (50% *v*/*v*), at which we obtained the maximum conversion. Although the conversion was moderate, some H_2_O_2_ was still present at the end of the reaction (after 60 min) as confirmed by adding an acidic potassium iodide solution to the reaction medium and observing the formation of iodine. This qualitative test ruled out that H_2_O_2_ could be the limiting agent. Furthermore, the addition of higher H_2_O_2_ concentrations did not further improve the conversion of styrene, but however over-oxidation byproducts (mainly benzoic acid) were formed, thus decreasing the selectivity towards benzaldehyde. The temperature also had a strong influence on the reaction; the higher the temperature, the better the conversion, achieving a maximum conversion at 120 °C. Taking into account that the boiling point of the solvent used (acetonitrile) is 82 °C, the application of pressure to the closed-vessel microwave sample was required to achieve a temperature of 120 °C. On the other hand, time was found to be a critical parameter since 60 min was required to achieve a conversion of 45%. Unfortunately, longer reaction times hardly improved the conversion, which varied only from 45% to 51% for 60 and 90 min, respectively). Thus, a maximum conversion of 45% may be explained due to a saturation of the active sites present on the catalyst surface.

In order to evaluate the reusability of the α-Fe_2_O_3_/RH-Silica catalyst, the solid material was separated from the reaction media after reaction, washed, dried, and reused for other run of the reaction. After three catalytic uses, the conversion and the selectivity remained without significant changes which indicated the great stability of the supported α-Fe_2_O_3_ nanoparticles in this reaction (Table 2, entries 3, 4, and 5). Additionally, no Fe leaching from the catalyst during the reaction was found, as determined by ICP-MS analysis (no detectable Fe into the solution, <0.2 ppm), which also confirmed the stability of the catalyst under the reaction conditions.

## 3. Materials and Methods

### 3.1. Obtaining Biosilica from Rice Husk Waste

Biogenic silica from rice husk (RH-Silica) was obtained by an integrated mechanochemical/microwave approach previously reported and patented by our group [23,39]. All chemicals used were of analytical grade and were used as received without any further purification and were obtained from Sigma-Aldrich. Rice husk was first milled with a Retsch PM 100 planetary ball milling (Retsch GmbH & Co. Kg, Haan, Germany), consisted of a 125 mL chamber with 18 stainless steel balls (10 mm diameter, 5 g weigh). Subsequently, a microwave-assisted acid digestion was performed by using an ETHOS-ONE multimode microwave (Milestione Inc., Shelton, CT, USA) in order to obtain a highly pure amorphous mesoporous silica. To carry out the extraction a mild acid, HCl solution (0.3 M) was added to 2 g of rice husk with the aim of extracting the silica from the rice husk, as well as removing the metal ions present in the rice husk. The extraction process was conducted at 300 W for 30 min. The resulted material was then filtered, washed with distilled water, dried at 100 °C for 24 h, and calcinated at 550 °C for 4 h to obtain finally a highly pure (>95%) biosilica.

### 3.2. Mechanochemical Synthesis of α-Fe_2_O_3_/RH-Silica

RH-Silica (1.27 g) was grinded in the same planetary ball milling described above, together with Fe(NO_3_)_3_·9H_2_O (3.40 g; Sigma-Aldrich #216828) as iron precursor and propionic acid (0.63 mL; Sigma-Aldrich #402907). The mechanochemical process was carried out at optimized conditions (350 rpm, 10 min). Afterwards, Fe_2_O_3_/RH-Silica was heated up (1 °C·min^−1^) to at 300 °C and remained at this temperature for 4 h to ensure that all iron precursor was transformed to iron oxide NPs. Moreover, due to this thermal treatment the iron oxide NPs were transformed to hematite phase (α-Fe_2_O_3_), the most thermically stable phase.

### 3.3. Material Characterization

Materials were characterized by multiple techniques including N_2_ physisorption, powder X-Ray Diffraction (XRD), Diffuse Reflectance Infrared Transform spectroscopy (DRIFT), Scanning Electron Microscopy (SEM), and Electron Transmission Microscopy (TEM). N_2_ physisorption was carried out in a Micromeritics ASAP adsorption analyzer (Micromeritics Corp., Norcross, GA, USA) at 77 K. Samples were previously degassed under vacuum at 130 °C for 24 h. Materials surface areas were calculated by Brunauer-Emmet-Teller equation (BET) while pore size and pore diameter were obtained from Barret-Joyner-Halenda (BJH) model. XRD spectra were recorded in a Bruker D8 diffractometer (Bruker Corp., Billerica, MA, USA) operating at 40 kV and 40 mA, and using CuK_α_ (λ = 0.15406) as radiation. SEM and TEM images were obtained in a JEOL-SEM JSM-6620 and a JEM 2010F (JEOL GmbH, Freising, Germany), respectively. DRIFT spectra of absorbed pyridine were collected in a PIKE Technologies MB3000-PH FT-IR spectrometer (ABB Inc., Québec, QC, Canada) at 100 °C, 150 °C, 200 °C, and 300 °C. Before analysis, samples absorbed pyridine for 30 min to achieve complete saturation. To determine the amount of Fe incorporated and the potential Fe leaching during reaction, inductively coupled plasma mass spectrometry (ICP-MS) analysis were performed by using an ELAN DRC-e ICP-MS (PerkinElmer SCIEX, Billerica, MA, USA). The digestion of the samples was carried out overnight with a mixture of 1:1:1 HF/HNO_3_/HCl acids.

### 3.4. Catalytic Activity

The selective oxidation of styrene was carried out under microwave irradiation in a CEM-Discover reactor (CEM Corporation, Matthews, NC, USA). To perform the microwave-assisted reaction under optimized conditions, acetonitrile (ACN, 2 mL), styrene (0.2 mL), H_2_O_2_ (50% *v*/*v*, 0.3 mL), and the catalyst (0.05 g) were placed in a closed vessel (pressure controllable) and heated at 120 °C during 60 min under continuous stirring. The microwave power was set up at 150 W in order to achieve the desired temperature, which was measured by an infrared probe. After reaction, the reaction mixture was filtered and analyzed by gas chromatography (GC) in an Agilent 6890N chromatograph (Agilent Technologies Inc., Wilmington, DE, USA) fitted with an HP-5 capillary column (30 m × 0.32 mm × 0.25 μm) and equipped with a flame ionization detector (GC-FID). Additionally, samples were analyzed by GC-MS to identify the products obtained. Conversion of styrene and selectivity to benzaldehyde were calculated as follows:

$$\mathrm{Conversion}\;\left(\%\right)=\frac{C_{o,styrene}-C_{styrene}}{C_{o,DMF}}\times 100$$

$$\mathrm{Selectivity}\;\left(\%\right)=\frac{C_{benzaldehyde}}{C_{o,styrene}-C_{styrene}}\times 100$$

where *C_o_* and *C* correspond to initial and final molar concentrations in the reaction mixture quantified by GC-FID.

## 4. Conclusions

An efficient iron oxide nanoparticle-based catalyst (α-Fe_2_O_3_/RH-Silica) was synthesized by mechanochemistry using rice husk-derived mesoporous biosilica as a green inorganic support. The obtained material, consisted of iron oxide NPs as pure hematite phase immobilized on the biosilica, displayed a remarkable Lewis acidity even at high temperatures. These relevant properties were exploited to produce a high-added value compound, i.e., benzaldehyde, from the oxidation of styrene under microwave irradiation. It is important to remark that a 100% selectivity towards the desired product was obtained, together with an acceptable yield of 45% in only 1 h reaction time. Moreover, the reusability of the catalyst was successfully demonstrated. This work serves to illustrate the potential of using biomass-derived inorganic supports for the preparation of efficient catalysts as well as their feasibility to be applied in industrial relevant processes as, for example, in the production of benzaldehyde.

## Figures and Tables

**Table 1:** Textural properties of RH-Silica and α-Fe_2_O_3_/RH-Silica materials.

Material	S_BET_^a^ (m^2^·g^−1^)	D_BJH_^b^ (nm)	V_BJH_^c^ (cm^3^·g^−1^)
RH-Silica	352	8.0	0.56
α-Fe_2_O_3_/RH-Silica	272	7.1	0.39

^a^ S_BET_: specific surface area was calculated by the Brunauer-Emmet-Teller (BET) equation. ^b^ D_BJH_: mean pore diameter was calculated by the Barret-Joyner-Halenda (BJH) equation. ^c^ V_BJH_: pore volume was calculated by the Barret-Joyner-Halenda (BJH) equation.

**Table 2:** Microwave-assisted oxidation of styrene using α-Fe_2_O_3_/RH-Silica as catalyst ^a^.

Entry	Material	Conversion	Selectivity to Benzaldehyde	Selectivity to Others
		(mol%)	(mol%)	(mol%)
1	-	-	-	-
2	RH-Silica	-	-	-
3	α-Fe_2_O_3_/RH-Silica	45	>99	-
4	Reuse 1	43	>99	-
5	Reuse 2	44	>99	-

^a^ Reaction Conditions: 2 mL Acetonitrile (ACN), 0.2 mL Styrene, 0.3 mL H_2_O_2_ (50% *v*/*v*), 50 mg catalyst, 60 min, 120 °C, 100 PSI, 150 W.
